# Prevalence of polypharmacy and factors impacting psychotropic prescribing patterns in women of childbearing potential at inpatient mental health services in Qatar

**DOI:** 10.1002/npr2.12467

**Published:** 2024-08-20

**Authors:** Nervana Elbakary, Oraib Abdallah, Sami Ouanes, Ahmad Hasanoglu, Eiman Abedlfattah‐Arafa, Maha Al‐Shaikhly, Shatha Alqam, Sulaiman Alshakhs, Zainab Hijawi, Majid Al‐Abdulla, Noriya Al‐Khuzaei, Sazgar Hamad

**Affiliations:** ^1^ Pharmacy Department, Mental Health Services Hamad Medical Corporation Doha Qatar; ^2^ Medical Department, Mental Health Services Hamad Medical Corporation Doha Qatar; ^3^ Pharmacy Department AlKhor Hospital, Hamad Medical Corporation Doha Qatar

**Keywords:** antipsychotics, chlorpromazine, hospital stay, suicidal history, treatment response

## Abstract

**Aims:**

Women may experience unique mental disorders due to hormone shifts. Rates of schizophrenia and bipolar disorder are similar between genders, but onset and symptoms may differ. Women tend to use more psychotropic drugs due to limited therapeutic options. This study was aimed to estimate the prevalence of psychotropic polypharmacy among females of childbearing potential and factors impacting prescribing patterns.

**Methods:**

This was a quantitative retrospective chart review for patients admitted to inpatient units at the Mental Health Hospital in Qatar. SPSS® Statistics was used for data analysis. In addition to descriptive statistics applied, linear regression and binary logistic regression models were used to examine the clinical and sociodemographic factors associated with polypharmacy and full therapeutic response upon discharge, respectively. An alpha value of 0.05 was used.

**Results:**

Of the 347 patients, 52.7% of the patients received a prescription of at least two psychotropic drugs upon discharge. Around two‐thirds (63.1%) were prescribed at least one antipsychotic. Potential predictors of polypharmacy were age (*p* = 0.027), longer hospital stay (*p* = 0.003), family history (*p* < 0.001), absence of suicidal history (*p* = 0.005), and a diagnosis of a mood disorder (*p* = 0.009), or a diagnosis of a psychotic disorder (*p* = 0.015). A full response upon discharge was less likely to occur in patients with a longer stay (OR = 0.940; *p* = 0.029) and in those with a substance use disorder (OR = 0.166; *p* = 0.035).

**Conclusion:**

There is a notably high prevalence of total polypharmacy upon discharge. Some identified factors are modifiable. Evidence‐based prescription practices through hospital guidelines and education should be emphasized to avoid unreasonable polypharmacy.

## Abstract 2

This research offers a comprehensive review of polypharmacy prevalence and psychotropic prescribing patterns among women of childbearing potential in Qatar's mental health services. We identify numerous factors, both modifiable and unmodifiable, influencing polypharmacy, advancing understanding in psychiatry and women's health across the Middle East and beyond. The paper endorses the implementation of rationalized, evidence‐based prescription practices customized to meet the specific requirements of female psychiatric patients.

## Graphical abstract

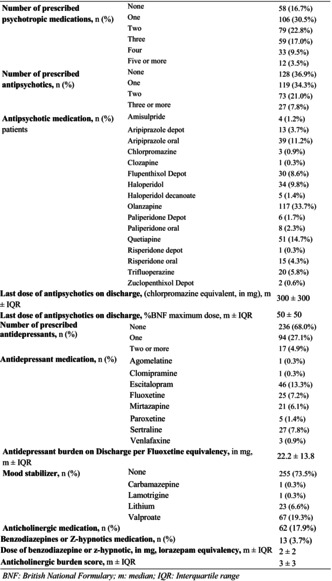

## 1 INTRODUCTION

Mental disorders can affect women and men differently. Some disorders are more prevalent in women such as depression and anxiety.^1^ There are also certain types of mental disorders that are unique to women; especially at times of hormone shifts, such as perinatal depression, premenstrual dysphoric disorder, and perimenopause‐related depression.^2^ When it comes to other mental disorders such as schizophrenia and bipolar disorder, research has not found significant differences in the rates at which men and women experience these illnesses.^3^, ^4^ However, onset of the illness as well as certain symptoms may occur more frequent in women than in men, and the course of the illness itself can be affected by the biological differences in sex.^5^ Previous studies have shown increasing rates of psychotropic use, polypharmacy, and off‐labelling use among women rather than men, especially if other categories of therapeutic interventions are not available or accessible.^6^

Globally, the prescribing pattern of psychotropics is different between geographical areas and is influenced by many factors like patient characteristics, genetics, diagnosis, cultural factors, socioeconomic status, availability of newer drugs in the local market or formulary and prescribing habits of physicians.^7^, ^8^ Additionally, international agencies like the World Health Organization (WHO) and the International Network for Rational Use of Drugs (INRUD) have recommended standard drug use indicators which can help health facilities' managers and policy makers to monitor therapeutic trends and to identify the factors responsible for the usual practice of polypharmacy, off‐label use of medications and the health hazards associated with these practices such as adverse drug effects (ADEs).^9^, ^10^ Therefore, tracking prescribing behavior, measurement of drug use and giving suggestions, if necessary, about the modifications needed to make medical care safe, rational, and cost effective is paramount.^11^

Many studies from the Western countries have reported the prescription patterns of various psychotropic medications, which have investigated national prescription patterns. Some have investigated prescription patterns in general adult psychiatry, and few reports specialized in individual exposures to certain categories of psychotropic medications such as antidepressants, antipsychotics, mood stabilizers in bipolar disorder patients, and rate of use of anticholinergic agents in psychiatric patients.^12^, ^13^ Regionally, fewer articles of psychotropic prescription patterns from Middle East are available in the literature with special interest in prescribing pattern of psychotropics in women psychiatry and the extent of suitability of medication use for women in the childbearing age.

We aimed to prioritize women in our research based on the findings of the epidemiological study conducted by Bener, et al, that examined the prevalence and burden of psychiatric disorders in primary health care visits in Qatar and found that the prevalence in women was significantly higher than men for the most common psychiatric disorders, specifically generalized anxiety disorder, panic disorder, social phobia, specific phobias, obsessive compulsive disorders, post‐traumatic disorder, somatization, major depressive disorder, bipolar disorder, dysthymia, and oppositional defiant disorder.^14^

Moreover, this emphasis on women stems from the distinctive factors associated with potential differences in the pharmacokinetics/pharmacodynamics of drugs in relation to women as opposed to men such as the higher susceptibility to adverse effects such as hair loss, acne, and hyperprolactinemia, and serious implications for pregnancy and breastfeeding. Notably, prior studies on prescription patterns in Qatar generally overlooked the specific focus on women and instead concentrated on antipsychotics use. For instance, a study done by Bastaki et al, assessed the prescription pattern and off‐label use of antipsychotics in the Qatari population and found that among the 9349 Qatari patients prescribed antipsychotics during the study period, the majority were females and were in the age category between 30 and 39 years (57%).^6^ Also, S. Ouanes et al, investigated the factors that was associated with antipsychotics polypharmacy in Qatar, and his model showed that antipsychotic polypharmacy was independently associated with the female gender after controlling for other factors (*p* = 0.041; OR = 6.52 with 95% CI: 1.07–39.61).^15^

Polypharmacy, as outlined in the 2001 Technical Report by the National Association of State Mental Health Program Directors (NASMHPD), encompasses different types. First, same‐class polypharmacy is defined by the concurrent use of multiple medications from the same class, for example: the simultaneous prescriptions of two atypical antipsychotics for treatment of schizophrenia. Second, multiclass polypharmacy implies ordering full therapeutic doses of medications from different classes to address an overlapped symptom cluster, such as combining valproate and atypical antipsychotics for the management of mania induced by another from a different class, like using trazodone to address insomnia resulting from the use of bupropion. Augmentation polypharmacy, the third category, includes scenarios where a subtherapeutic dose of one medication is used alongside a different class medication at its full therapeutic for the same symptom cluster, or the addition of a medication not used alone for the same purpose, as augmenting antidepressants with lithium or thyroid hormone. Finally, total polypharmacy refers to the overall count of medications prescribed or the total psychotropics burden.^16^, ^17^

Hence, as there were no previous data tailored specifically about female psychiatric patients with emphasis on the diversity of psychiatric disorders and medication use across their lifespan like pregnancy, postpartum, and perimenopausal periods. Our study endeavors to estimate the prevalence of psychotropic drug categories and understand their prescribing patterns among females of childbearing potential. We specifically aim to explore the impact of polypharmacy during inpatient admission period or hospitalization. Through a systematic retrospective database mining approach, we have analyzed polypharmacy trends and types. Furthermore, our investigation seeks to examine the sociodemographic and clinical features associated with polypharmacy, with a focus on its implications on side effects and risks of medication, especially in the childbearing years.

## 2 METHODS

### 2.1 Ethical considerations

The study was conducted in Qatar with the approval of the Institutional review board of Medical Research Center at Hamad Medical Corporation with reference number: MRC‐01‐22‐752 and it conforms to the provisions of the Declaration of Helsinki. No participation consent was obtained due to the data being retrospectively collected. Participants were identified by using codes (i.e. WWI001). The link between code and identifier was deleted at the end of the study and information collected was kept confidential and reported as group data.

### 2.2 Study design, participants, and study setting

This was a quantitative retrospective chart review of patient medical files using Cerner®. The study was conducted at Mental Health Services in Qatar, the primary provider of psychiatric care for patients diagnosed with mental illnesses in the country. The hospital offers inpatient and outpatient services for various populations, including children, adolescents, perinatal individuals, general adults, and older adults. Data collection focused on the inpatient facilities for females, comprising two acute units. The first, located at the MHH building, served as the main acute care unit for females, featuring 17 beds with an occupancy rate exceeding 95%. The second unit was the Women Wellness ward at Bayt al‐Dyiafa, which had 12 beds at the time of planning the study. The study involved the review of chart notes of patients who were admitted to these two units at the Mental Health Hospital in Qatar. We included women of childbearing age within the range of 16–55 years old, while exclusion criteria applied to women of childbearing age who, despite having contact with the service as an inpatient, were not prescribed any psychotropic medications.

### 2.3 Sampling method and sample size determination

To calculate the sample size in the absence of reported prevalence from Qatar in the literature, the expected proportion in the population on polypharmacy (*p*) was assumed to be 25%.^18^ The absolute error or precision (*d*) was specified as 0.05 and alpha value was set as 0.05. Using these parameters, the calculated sample size amounted to 288 patients. Anticipating potential missing data, sample size was inflated by 20%, resulting in a final sample size of 346 patients selected through a simple random sampling technique.

### 2.4 Study instruments

A tool has been developed to collect all necessary data inclusive of the period (from Jun 2021 to Jun 2022) and no data beyond June 2022 was collected. Data were collected at one point only. The tool encompassed demographics such as age, marital status, and reproductive stage. Additionally, clinical variables including pregnancy status, breastfeeding status, and diagnosis were captured. The tool also incorporated medication‐related variables, such as the type of medications and detailed regimens, identification of drug–drug interactions if any, documentation of adverse drug events, and details regarding the number of admissions and the duration of illness. The diagnosis of illness stated in this paper are based on criteria outlined in The Diagnostic and Statistical Manual of Mental Disorders, Fifth Edition DSM‐5.^19^ Response to therapy is usually defined as per established criteria for treatment response for each illness (i.e: Hamilton Depression Rating Scale (HAM‐D) for MDD and Positive and Negative Syndrome Scale (PANSS) for schizophrenia).^20^, ^21^ However, in the absence of routine use of detailed and specific psychometric scales in a naturalistic study like ours, response was defined based on the global clinical impression taking into account the scales above. The development of this tool underwent a rigorous process, involving revision by three members of the group and testing on ten patient profiles to ensure its appropriateness, clarity, and effectiveness in capturing the required information.

### 2.5 Statistical analysis

The Statistical Package for the Social Sciences (SPSS) software (IBM SPSS® Statistics for Windows; IBM Corp, Armonk, New York, USA) version 26 was used for data analysis. We determined absolute and relative frequencies for categorical variables. Since none of our continuous variables followed a normal distribution (as per Shapiro‐Wilk's test), we determined the median and the interquartile range (IQR) for each of these variables.

To examine the clinical and sociodemographic factors associated with polypharmacy, we constructed a linear regression using the number of psychotropic medications as a dependent variables and the following clinical and sociodemographic characteristics as independent variables (using the enter method): age, nationality (Qatari vs. non‐Qatari), length of stay, discharge unit, occupational status (employed or studying vs. unemployed), family history of psychiatric illness, duration of illness (> = 1 year vs. <1 year), somatic comorbidity, suicidal history (thoughts or attempt), the presence of a mood disorder, the presence of a psychotic disorder, the presence of a substance use disorder, the presence of a personality disorder, and the presence of an anxiety disorder, OCD or PTSD. We determined the unstandardized regression coefficients (*B*), their 95% confidence intervals (CIs), and the partial Spearman's Coefficients (*r*). We also calculated the variance inflation factor (VIF) to check for multicollinearity.

To examine the clinical and sociodemographic factors associated with a full therapeutic response upon discharge, we constructed a binary logistic regression model. The therapeutic response was dichotomized (full response vs. partial or no response) and used as a dependent variable. The following clinical and sociodemographic characteristics were entered as independent variables: age, nationality (Qatari vs. non‐Qatari), length of stay, discharge unit, occupational status (employed or studying vs. unemployed), family history of psychiatric illness, duration of illness (> = 1 year vs. <1 year), somatic comorbidity, suicidal history (thoughts or attempt), the presence of a mood disorder, the presence of a psychotic disorder, the presence of a substance use disorder, the presence of a personality disorder, and the presence of an anxiety disorder, OCD or PTSD, the number of psychotropic medications. We calculated odds ratios (ORs) and 95% CIs. For all analyses, an alpha‐value of 0.05 was used.

## 3 RESULTS

### 3.1 Sociodemographic and clinical characteristics

We included 347 female patients. The median age was 30 years with an IQR of 12. The most important nationality groups consisted of Qataris (24.0%, *n* = 83), non‐Qatari Arabs (25.1%, *n* = 87), and Southeast Asians (22.8%, *n* = 79). Around one third (31.7%, *n* = 110) had a medical comorbidity, the most common of which were diabetes mellitus (13.3%, *n* = 43), thyroid disease (8.7%, *n* = 28), and hypertension (7.1%, *n* = 23). The median length of inpatient stay was 13 days with an IQR of 10. The most common disorders consisted of bipolar disorder (19.8%, *n* = 80), depressive disorder (15.8%, *n* = 64), personality disorder (10.6%, *n* = 43), and adjustment disorder (10.1%, *n* = 41). Upon discharge, 77.4% (*n* = 240) had a full response (Table 1).

**TABLE 1:** Sociodemographic and clinical characteristics.

Age (years), m ± IQR	30.0 ± 12.0
Nationality group, *n* (%)	
Qatari	83 (24.0%)
Arab & non‐Qatari	87 (25.1%)
Indian Subcontinent	54 (15.6%)
Southeast Asia	79 (22.8%)
Europe	2 (0.6%)
Sub‐Saharan Africa	36 (10.4%)
Other	5 (1.4%)
Marital status, *n* (%)	
Single	81 (49.1%)
Married	83 (50.3%)
Divorced	1 (0.6%)
Discharge unit, *n* (%)	
Acute female psychiatry unit	220 (63.6%)
Women's wellness unit	126 (36.4%)
Length of inpatient stay (days), m ± IQR	13.0 ± 10.0
Occupation, *n* (%)	
Unemployed	140 (44.3%)
Employed	131 (41.5%)
Student	45 (14.2%)
Duration of illness, *n* (%)	
Less than 1 year	102 (33.2%)
Between 1 and 5 years	133 (43.3%)
Between 6 and 10 years	49 (16.0%)
More than 10 years	23 (7.5%)
Number of lifetime admission to psychiatry, *n* (%)	
0	7 (2.3%)
1	170 (55.6%)
2	60 (19.6%)
3	28 (9.2%)
4 or more	41 (13.4%)
History of suicide ideation or attempt, *n* (%)	160 (49.5%)
Diabetes, *n* (%)	43 (13.3%)
Hypertension, *n* (%)	23 (7.1%)
Dyslipidemia, *n* (%)	10 (3.1%)
Cancer, *n* (%)	0 (0%)
Coronary disease, *n* (%)	1 (0.3%)
Cerebrovascular disease, *n* (%)	1 (0.3%)
DVT/PE, *n* (%)	0 (0%)
Renal disease, *n* (%)	0 (0%)
Liver disease, *n* (%)	0 (0%)
Thyroid disease, *n* (%)	28 (8.7%)
Smoking, *n* (%)	
Current smoker	30 (8.9%)
Ex‐smoker	5 (1.5%)
Never smoked	233 (69.1%)
Alcohol use, *n* (%)	39 (12.1%)
Use of other substances, *n* (%)	15 (4.7%)
Family history of psychiatric illness, *n* (%)	40 (12.7%)
Psychiatric diagnosis, *n* (%)	
No mental illness	28 (6.9%)
Bipolar disorder	80 (19.8%)
Depressive disorder	64 (15.8%)
Personality disorder	43 (10.6%)
Adjustment disorder	41 (10.1%)
Schizophrenia	26 (6.4%)
Schizoaffective disorder	25 (6.2%)
Schizophreniform/brief psychotic disorder	25 (6.2%)
Substance use disorder	12 (3.0%)
Intellectual disability	11 (2.7%)
Other anxiety disorders	9 (2.2%)
Delusional disorder	5 (1.2%)
OCD and related disorders	5 (1.2%)
Other	30 (7.4%)
Treatment outcome, *n* (%)	
Full response	240 (77.4%)
Partial response	66 (21.3%)
No response	4 (1.3%)

Abbrevitions: IQR, interquartile range; m, median.

### 3.2 Prescription patterns

Around half (52.7%, *n* = 183) of the patients received a prescription of at least two psychotropic drugs upon discharge. Around two‐thirds (63.1%, *n* = 219) were prescribed at least one antipsychotic and around one third (32.0%, *n* = 111) were prescribed at least one antidepressant. In addition, 26.5% (*n* = 92) were given a mood stabilizer and 17.9% (*n* = 62) an anticholinergic drug.

Among patients who were prescribed an antipsychotic drug, 45.6% (*n* = 100) were on antipsychotic polypharmacy. The most prescribed antipsychotic medication was olanzapine followed by quetiapine then aripiprazole (Table 2). The median antipsychotic dose on discharge was 300 mg in chlorpromazine equivalency with an IQR of 300 and a range between 25 and 1500 mg.

**TABLE 2:** Prescription patterns.

Number of prescribed psychotropic medications, *n* (%)	
None	58 (16.7%)
One	106 (30.5%)
Two	79 (22.8%)
Three	59 (17.0%)
Four	33 (9.5%)
Five or more	12 (3.5%)
Number of prescribed antipsychotics, *n* (%)	
None	128 (36.9%)
One	119 (34.3%)
Two	73 (21.0%)
Three or more	27 (7.8%)
Antipsychotic medication, *n* (%) patients	
Amisulpride	4 (1.2%)
Aripiprazole depot	13 (3.7%)
Aripiprazole oral	39 (11.2%)
Chlorpromazine	3 (0.9%)
Clozapine	1 (0.3%)
Flupenthixol Depot	30 (8.6%)
Haloperidol	34 (9.8%)
Haloperidol decanoate	5 (1.4%)
Olanzapine	117 (33.7%)
Paliperidone Depot	6 (1.7%)
Paliperidone oral	8 (2.3%)
Quetiapine	51 (14.7%)
Risperidone depot	1 (0.3%)
Risperidone oral	15 (4.3%)
Trifluoperazine	20 (5.8%)
Zuclopenthixol Depot	2 (0.6%)
Last dose of antipsychotics on discharge, (chlorpromazine equivalent, in mg), m ± IQR	300 ± 300
Last dose of antipsychotics on discharge, %BNF maximum dose, m ± IQR	50 ± 50
Number of prescribed antidepressants, *n* (%)	
None	236 (68.0%)
One	94 (27.1%)
Two or more	17 (4.9%)
Antidepressant medication, *n* (%)	
Agomelatine	1 (0.3%)
Clomipramine	1 (0.3%)
Escitalopram	46 (13.3%)
Fluoxetine	25 (7.2%)
Mirtazapine	21 (6.1%)
Paroxetine	5 (1.4%)
Sertraline	27 (7.8%)
Venlafaxine	3 (0.9%)
Antidepressant burden on discharge per fluoxetine equivalency, in mg, m ± IQR	22.2 ± 13.8
Mood stabilizer, *n* (%)	
None	255 (73.5%)
Carbamazepine	1 (0.3%)
Lamotrigine	1 (0.3%)
Lithium	23 (6.6%)
Valproate	67 (19.3%)
Anticholinergic medication, *n* (%)	62 (17.9%)
Benzodiazepines or Z‐hypnotics medication, *n* (%)	13 (3.7%)
Dose of benzodiazepine or z‐hypnotic, in mg, lorazepam equivalency, m ± IQR	2 ± 2
Anticholinergic burden score, m ± IQR	3 ± 3

Abbreviations: BNF, British National Formulary; IQR, interquartile range; m, median.

Among patients who were prescribed an antidepressant drug, only 15.3% (*n* = 17) had more than one antidepressant. The most common antidepressant medications were escitalopram, sertraline, fluoxetine, and mirtazapine (Table 2). The median antidepressant dose (in fluoxetine equivalent mg) was 22.2 with an IQR of 13.8. Among mood stabilizers, the most prescribed drug was valproate (*n* = 67) followed by lithium (*n* = 23).

### 3.3 Sociodemographic and clinical characteristics associated with polypharmacy

The total number of prescribed psychotropic drugs on discharge was higher in older patients (*B* = 0.020; *p* = 0.027; *r* = 0.135), patients with a longer inpatient stay (*B* = 0.038; *p* = 0.003; *r* = 0.178), those with a positive psychiatric family history (*B* = 0.890; *p* < 0.001; *r* = 0.239), a negative suicidal history (*B* = −0.426; *p* = 0.005; *r* = −0.171), a diagnosis of a mood disorder (*B* = 0.467; *p* = 0.009; *r* = 0.159), or a diagnosis of a psychotic disorder (*B* = 0.518; *p* = 0.015; *r* = 0.148). Other sociodemographic (age, citizenship, occupation) or clinical variables (discharge unit, duration of illness, somatic comorbidity, the presence of a substance use disorder, and that of an anxiety disorder or OCD or PTSD) were not found to be associated with polypharmacy (Table 3).

**TABLE 3:** Multiple linear regression model with the number of psychotropic drugs upon discharge as a dependent variable and clinical and sociodemographic variables as independent variables.

Variable	*B*	*p*	95.0% confidence interval for *B*		Partial regression	VIF
			Lower bound	Upper bound		
Age	**0.020**	**0.027**	**0.002**	**0.037**	**0.135**	**1.573**
Citizenship (Qatari vs. non‐Qatari)	0.048	0.788	−0.306	0.403	0.016	1.264
Length of stay	**0.038**	**0.003**	**0.013**	**0.063**	**0.178**	**1.170**
Discharge unit	0.269	0.065	−0.017	0.556	0.112	1.099
Occupational status (employed or studying vs. unemployed)	−0.256	0.121	−0.579	0.068	−0.094	1.473
Family history of psychiatric illness	**0.890**	**0.000**	**0.456**	**1.323**	**0.239**	**1.088**
Duration of illness (>=1 year vs. <1 year)	0.252	0.129	−0.074	0.577	0.092	1.332
Somatic comorbidity	−0.213	0.177	−0.523	0.097	−0.082	1.219
Suicidal history	**−0.426**	**0.005**	**−0.722**	**−0.131**	**−0.171**	**1.235**
Mood disorder	**0.467**	**0.009**	**0.119**	**0.815**	**0.159**	**1.681**
Psychotic disorder	**0.518**	**0.015**	**0.102**	**0.933**	**0.148**	**1.859**
Substance use disorder	−0.255	0.558	−1.111	0.601	−0.036	1.133
Personality disorder	0.063	0.795	−0.416	0.543	0.016	1.569
Anxiety disorders, OCD or PTSD	−0.014	0.965	−0.642	0.614	−0.003	1.113

*Note*: Figures in bold font indicate statistical significance.

Abbreviations: OCD, obsessive compulsive disorder; PTSD, post‐traumatic stress disorder; VIF, variance inflation factor.

### 3.4 Sociodemographic and clinical characteristics associated with clinical outcome upon discharge

A full response upon discharge (as opposed to a partial response or no response) was less likely to occur in patients with a longer length of inpatient stay (OR = 0.940 [0.889; 0.994]; *p* = 0.029) and in those with a substance use disorder (OR = 0.166 [0.031;0.885]; *p* = 0.035). Other sociodemographic (age, citizenship, occupation) or clinical variables (discharge unit, family history of psychiatric illness, duration of illness, somatic comorbidity, suicidal history, the presence of a psychotic disorder, of a mood disorder, or of an anxiety disorder or OCD or PTSD, number of psychotropic drugs on discharge) were not found to be associated with the therapeutic outcome (Table 4).

**TABLE 4:** Binary logistic regression model with the therapeutic outcome upon discharge as a dependent variable (full response vs. partial or no response) and clinical and sociodemographic variables as independent variables.

Variable	Wald	OR	95.0% confidence interval for OR		*p*
			Lower bound	Upper bound	
Age	0.067	0.995	0.958	1.033	0.796
Citizenship (Qatari vs. non‐Qatari)	0.023	0.943	0.443	2.010	0.880
Length of stay	**4.771**	**0.940**	**0.889**	**0.994**	**0.029**
Discharge unit	1.417	1.482	0.775	2.831	0.234
Occupational status (employed or studying vs. unemployed)	0.359	1.246	0.607	2.559	0.549
Family history of psychiatric illness	0.577	1.463	0.548	3.903	0.448
Duration of illness (> = 1 year vs. <1 year)	0.206	0.840	0.395	1.786	0.650
Somatic comorbidity	0.216	1.179	0.589	2.359	0.642
Suicidal history	0.383	1.232	0.636	2.386	0.536
Mood disorder	0.081	0.888	0.394	2.002	0.775
Psychotic disorder	0.065	1.132	0.437	2.930	0.798
Substance use disorder	**4.422**	**0.166**	**0.031**	**0.885**	**0.035**
Personality disorder	0.050	1.140	0.359	3.626	0.824
Anxiety disorders, OCD or PTSD	1.170	0.502	0.144	1.749	0.279
Number of drugs	2.162	0.827	0.642	1.065	0.141

*Note*: Figures in bold font indicate statistical significance.

Abbreviations: OCD, obsessive compulsive disorder; OR, odds ratio; PTSD, post‐traumatic stress disorder.

## 4 DISCUSSION

To our knowledge, this is the only recent study that assess prescribing patterns of all psychotropics in female inpatient units in Middle East at least. Our findings revealed a notably high prevalence of total polypharmacy upon discharge. Approximately 52.7% of patients were prescribed a combination of at least two psychotropic medications. Antipsychotics were the most used class, with 63.1% receiving at least one, and 45.6% of those prescribed antipsychotics were on polypharmacy. Additionally, 32.0% of patients were prescribed at least one antidepressant, and only 15.3% of those on antidepressants received more than one.

The prevalence of antipsychotic polypharmacy in this study was higher than the prevalence reported worldwide and by countries individually like Ethiopia, south Africa, Bahrain and Jordan.^18^, ^22^, ^23^, ^24^, ^25^ However, it is lower compared to the data published from Qatar in 2015 (Reported to be 59.3%).^26^ This significant variability in the prevalence of psychotropic polypharmacy across different regions depends on duration of studies, demographics of patients and treatment duration. It's important to highlight that the concept of polypharmacy is not universally perceived in a negative light. Some research indicates positive outcomes, such as a decrease in negative symptoms through the augmentation of aripiprazole.^27^ While the benefits of having more than two medications are not always certain and combinational strategies may put patients at risk of drug–drug interactions and heightened adverse drug reactions, some instances of polypharmacy align with established guidelines or are considered first‐line treatments for specific conditions.

The low prevalence of clozapine in our study suggests its underutilization in Qatar despite being a standard treatment for severe psychiatric conditions like refractory schizophrenia. Factors such as restricted access, stringent monitoring, and alternative therapies likely contribute to this trend.^28^

Mood stabilizers were given to 26.5% of patients, with valproate being the most frequently prescribed (19.3%). This finding raises concerns due to the known risks associated with valproate use in women in the childbearing age. Valproate has been associated with an increased risk of birth defects and developmental disorders in children born to mothers taking the medication during pregnancy.^27^, ^29^, ^30^ Given these serious potential risks, especially in the context of women of childbearing age, the prescription of valproate should be avoided unless no other alternative is available. Unfortunately, the perception of lithium, which is one of the first‐line treatments, as a “toxic drug” is common. This perception has led to significant underutilization by psychiatrists due to its side effects, such as thyroid dysfunction, renal dysfunction, and cognitive disturbance, which can be managed by early detection and close monitoring.^31^ The findings underscore the importance of careful consideration and communication between psychiatrists and patients regarding the potential risks and benefits of the use of valproate, particularly when it comes to those with reproductive implications. These data highlight the need for a hospital prescribing guideline.

In our study, the total number of prescribed psychotropic drugs on discharge was higher in older patients. This is supported by a meta‐analysis done in Africa where advanced age was associated with polypharmacy.^25^ Additionally, in another study approximately 60% of older patients prescribed a psychotropic drug in the prior year continued to receive such prescriptions in the following year. The most prevalent extended use was noted among patients aged 65 and older.^32^ Arnold et al. (2017) also reported that around 1.6 was the average of psychotropic medications prescribed per older adult patient at a minimum of one prescription.^33^ All of this indicate a substantial level of psychotropic drug use in older patients despite risk of falls, drug‐induced cognitive decline, and all‐cause mortality from excessive use of these medications.^34^, ^35^

Patients with an extended duration of inpatient stay received a higher total number of prescribed psychotropic drugs. The reason for this could be that patients with a longer inpatient stay often have more complex and/or more treatment‐resistant psychiatric conditions, leading psychiatrists to prescribe a more intensive prescription approach. Bressi et al reported that the presence of psychiatric comorbidity predicted longer hospital stays for medical inpatients and that mentally ill geriatric patients and those with schizophrenia or mood disorders were particularly at risk for longer lengths of stay.^36^

In a previous study, the likelihood of polypharmacy significantly increased when rescue treatments or as needed PRN treatments were considered. In the same study, polypharmacy was also more prevalent in the absence of family psychiatric history which oppose our finding that polypharmacy was higher in those with a positive psychiatric family history.^37^ Despite this, it is believed the shared experience of mental health challenges within a family may contribute to a higher likelihood of polypharmacy as the increased utilization of psychotropic polypharmacy in young individuals with parents experiencing multiple disabilities raises apprehensions regarding the oversight and monitoring of intricate psychotropic treatment.^38^

Surprisingly, we observed lower instances of polypharmacy among individuals with a suicidal history. This is not consistent with previous findings indicating that individuals with bipolar disorder for example faced a 64% likelihood of being prescribed complex polypharmacy if they had a history of suicide attempts. It is also noted that usually those with previous suicide attempt records are discharged with more than one drug prescription.^39^

The study did not find any associations between polypharmacy and substance use disorder, a finding that differs from that of Grau‐López et al concluded that patients diagnosed with a dual psychotic disorder were prescribed a larger number of medications compared to those with other psychiatric disorders.^40^

Our findings also suggest that a full response upon discharge is less likely to occur in patients with a longer length of inpatient stay and in those with a substance use disorder. It is expected that longer inpatient stay might indicate the complexity of the patient's condition. Patients with more severe mental health issues may require extended hospitalization, and achieving a full response in such cases could be challenging.^41^ Additionally, prolonged hospital stays might be associated with chronic or treatment‐resistant conditions, impacting the likelihood of a full response and does not always lead to better outcomes and may even be detrimental. In a review titled “The Optimal Length of Hospitalization for Psychiatric Patients,” the author examined various studies evaluating the value of different lengths of psychiatric hospitalization and alternatives to hospitalization the authors found important findings such as: firstly, longer stay doesn't decrease subsequent hospitalization. Secondly, limited improvement in social adjustment and psychopathology. In summary, the optimal length of psychiatric hospitalization should balance clinical needs with practical considerations, emphasizing effective aftercare planning to support patients beyond their hospital stay.^42^

Lastly, it is important to recognize that dual diagnosis—when an individual experiences both a mental health disorder and a substance use disorder simultaneously—can significantly complicate the treatment plan. These co‐occurring conditions create a complex interplay, where untreated mental health issues may exacerbate substance use problems and vice versa. As a result, dual diagnosis individuals often face an extensive treatment challenge for their psychiatric disorders, thus hindering a full response upon discharge.^43^, ^44^, ^45^

A notable strength of our research lies in being the first of its kind in the Middle East that provides insight into trends in prescribing patterns of psychiatric medications in hospitalized female patients only. In other words, studying the correlation between polypharmacy, treatment outcomes and other independent clinical features related to women distinguishes our research and adds a valuable dimension to the current body of knowledge. The implications of our findings extend beyond academic circles, holding relevance for policymakers and senior hospital managers. The insights gained from our research have the potential to guide strategic decisions about factors contributing to the length of stay, treatment response and to ensure rationalized approaches to women's care. By shedding light on prescribing patterns, our study seeks to mitigate drug‐related problems and reduce medication side effects, ultimately enhancing the overall quality of care for female psychiatric patients in Qatar and beyond.

While our findings provide valuable insights into prescribing trends, it is essential to acknowledge the limitations of our study. We did not study the appropriateness of prescription of the psychotropic drugs with regard to the diagnosis and co‐morbidities. Reproductive outcomes such as infertility, some obstetric and neonatal outcomes such as abortions or malformations, etc and prescribing in outpatient settings have not been included in this research, possibly we will elaborate on this point in separate research papers. The study was conducted in Qatar; hence findings cannot be easily extrapolated to other countries or regions. We also did not evaluate factors such as cost, patient consent to treatment, and prescribers' adherence to the international treatment guidelines. The study did not involve female patients admitted in drug addiction and rehabilitation centers. We did not analyze the use of PRNs given in the inpatient setting during the hospitalization, however, our assumption is that the more PRNs given for a patient, the more severity of illness and possibly the higher magnitude of polypharmacy. Additionally, the nature of our methodology as retrospective data utilization introduced issues of completeness and accuracy. Moreover, unmeasured confounding variables could have influenced observed associations. Future studies should try to overcome these limitations and investigate the unassessed measures.

## 5 CONCLUSION

In conclusion, our research provides a comprehensive overview of the prevalence of polypharmacy and factors impacting psychotropic prescribing patterns in women of childbearing potential at mental health services in Qatar. We highlighted many modifiable and unmodifiable factors that could be linked to the use of polypharmacy and contribute to the advancement of knowledge and practices in the field of psychiatry and women's health in the Middle East and beyond. This research also calls for action needed for more rationalized and evidence‐based prescribing practices that take into account the specific needs and preferences of female patients. We also suggest areas for future research that could further explore the appropriateness, safety, and cost‐effectiveness of psychotropic medications in this population, in addition to the association between polypharmacy and clinical outcomes, such as side effects.

## AUTHOR CONTRIBUTIONS

NE, OA, SO: Contribution to the idea conception, design of the study, data analysis, computation, and manuscript writing. AH, EM, MA, SHA, SUA, ZH: Contribution to study planning, data collection, manuscript writing. MA, NA, SH: Contributed to the study design and supervision of the project's overall progress. In addition to the contributions mentioned above, all authors revised the final manuscript critically for intellectual content and gave their valuable feedback and final approval of this version to be published.

## FUNDING INFORMATION

Open Access funding will be provided by the Qatar National Library.

## CONFLICT OF INTEREST STATEMENT

All authors declare that they have no competing interests.

## ETHICS STATEMENT

Approval of the research protocol by an Institutional Reviewer Board: This work was approved by the Medical Research Center (MRC) at Hamad Medical Corporation (Grant number: MRC‐01‐22‐752).

Informed Consent: N/A.

Registry and the Registration No. of the study: N/A.

Animal Studies: N/A.

## Supporting information


Data S1.

## Data Availability

The data that support the findings of this study are available in the supplementary material of this article.
